# Supplementary material for: Orally Administered Natural Lipid Nanoparticle-Loaded 6-Shogaol Shapes the Anti-Inflammatory Microbiota and Metabolome
Source: Pharmaceutics. 2021 Aug 28;13(9):1355. doi: 10.3390/pharmaceutics13091355 (PMC8472296; doi:10.3390/pharmaceutics13091355)
Supplement: Supplementary file 1 [file pharmaceutics-13-01355-s001.zip › pharmaceutics-1315912-supplementary-r3.pdf]

pharmaceutics

Supplementary Information: Orally administered natural lipid nanoparticle-loaded 6-shogaol shapes the anti-inflammatory microbiota and metabolome

Chunhua Yang, Dingpei Long, Junsik Sung, Zahra Alghoul and Didier Merlin

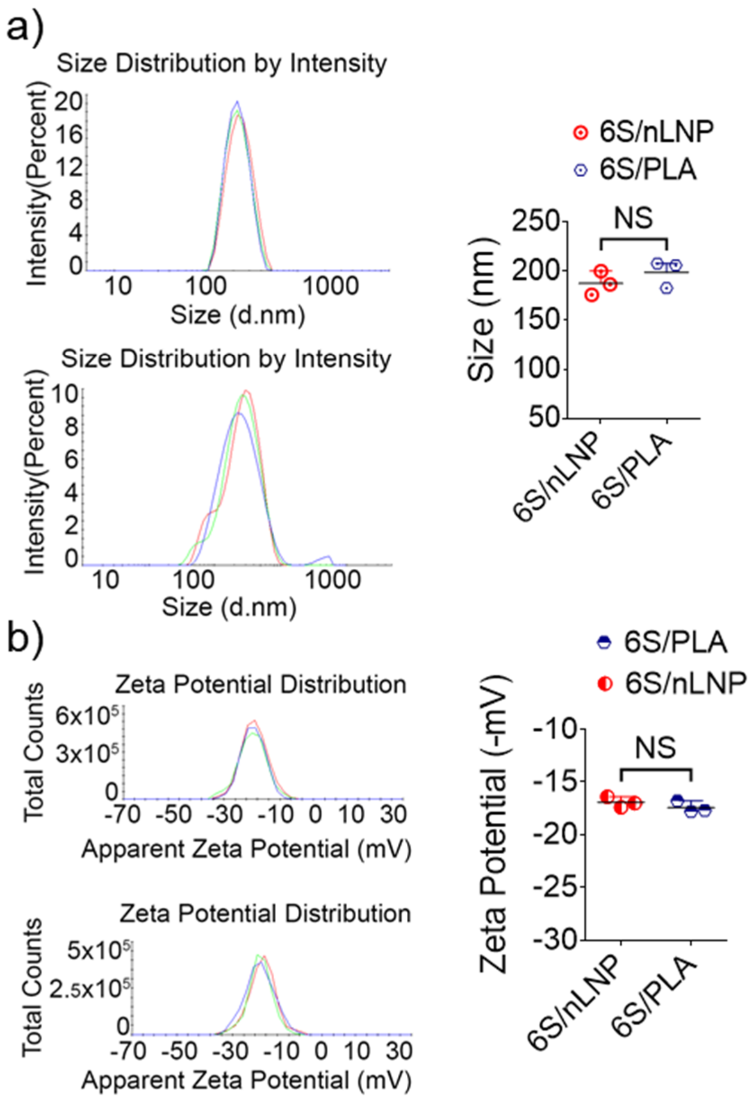

Figure S1. Size and zeta potential characterization of reassembled 6-shogaol loaded natural lipid nanoparticle (6S/nLNP) and 6-shogaol loaded PLA nanoparticles (6S/PLA). (**a**) Hydrated particle size distribution of 6S/nLNP (upper-left) and 6S/PLA (lower-left), and size comparison of 6S/nLNP versus 6S/PLA (right). (**b**) Zeta potential distribution of 6S/nLNP (upper-left) and 6S/PLA (lower-left), and zeta potential comparison of 6S/nLNP versus 6S/PLA (right).

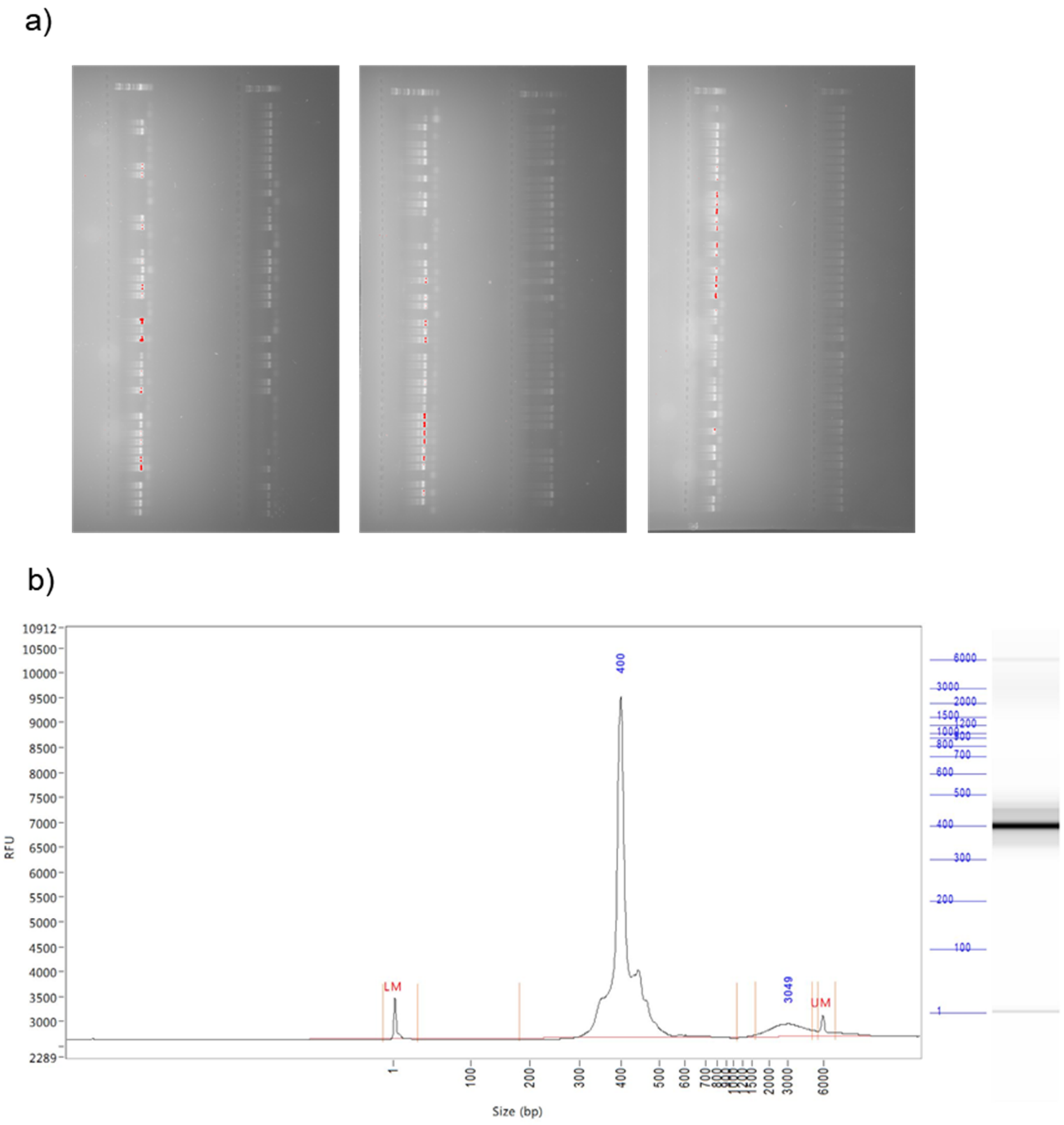

Peak	Size (bp)	Conc. (ng/uL)	From (bp)	To (bp)	RFU	Rel. Conc. % (ng/uL)	Molarity (nmole/L)
1	1 (LM)	0.2747	0	31	812		200.1279
2	400	80.3507	183	1088	6854	92.9	325.6197
3	3049	6.1716	1587	5096	259	7.1	3.2668
4	6000 (UM)	0.1258	5667	7132	416		0.0335

TIC:	86.5223	ng/uL
TIM:	328.8865	nmole/L
Total Conc.:	87.0833	ng/uL

Figure S2. Quality control (QC) of sample preparation for 16S rRNA sequencing. (**a**) Gel electrophoresis of RNA samples extracted from different groups of mouse feces, and (**b**) the quality analysis of final pooled RNA before 16S rRNA sequencing.

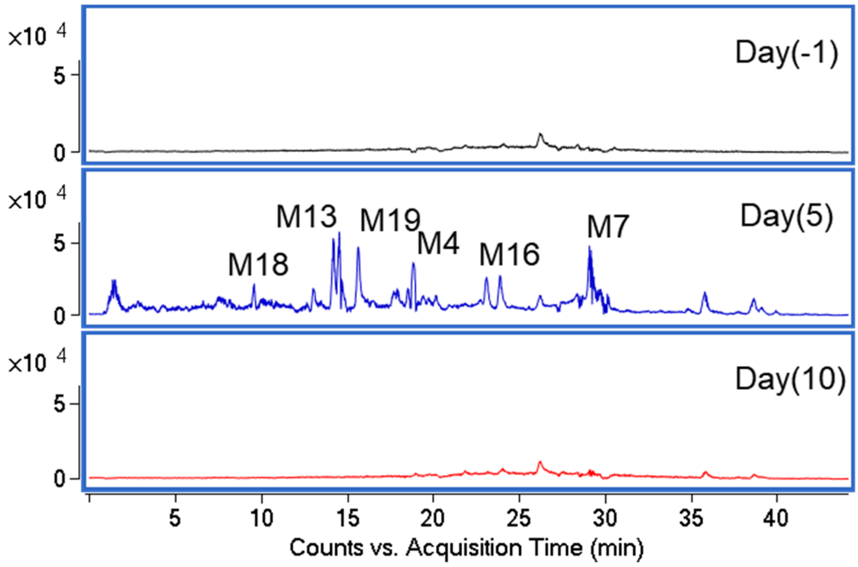

Figure S3. Extracted ion chromatogram (EIC, ESI+) LC-MS profiles of metabolites extracted from the different time points of 6S/nLNP treated mouse feces.

Metabolite profiles were extracted (by EIC or extracted ion chromatogram) from the total ion current chromatogram by utilizing an accurate molecular weight mass features extraction method. Metabolites used for accurate mass extraction were listed below (**Table S1**), and the mass error window was set at ± 5 ppm.

Table S1. Metabolites originated from 6-shogaol.

Compound	Formula	$[M+H]^+$	Compound	Formula	$[M+H]^+$
6S	$C_{17}H_{24}O_3$	277.1798	***M11***	$C_{17}H_{26}O_3$	279.1955
M1	$C_{20}H_{33}NO_5S$	400.2152	***M12***	$C_{18}H_{28}O_3S$	325.1832
M2	$C_{20}H_{31}NO_5S$	398.1996	***M13***	$C_{27}H_{41}N_3O_9S$	584.2636
M3	$C_{23}H_{35}N_2O_6S$	468.2289	***M14***	$C_{17}H_{22}O_3$	275.1642
M4	$C_{22}H_{35}NO_6S$	442.2258	***M15***	$C_{17}H_{24}O_3$	277.1798
M5	$C_{22}H_{33}NO_6S$	440.2101	***M16***	$C_{21}H_{31}NO_6S$	426.1945
M6	$C_{17}H_{26}O_3$	279.1955	***M17***	$C_{18}H_{26}O_4S$	339.1625
M7	$C_{18}H_{28}O_4$	309.2060	***M18***	$C_{17}H_{24}O_4S$	325.1468
M8	$C_{16}H_{24}O_3$	265.1798	***M19***	$C_{21}H_{33}NO_6S$	428.2101
M9	$C_{17}H_{28}O_3$	281.2111	***M20***	$C_{18}H_{28}O_4S$	341.1781
M10	$C_{18}H_{26}O_3S$	323.1675	***M21***	$C_{17}H_{26}O_4S$	327.1625
